# Mobile Robot Positioning with Wireless Fidelity Fingerprinting and Explainable Artificial Intelligence

**DOI:** 10.3390/s24247943

**Published:** 2024-12-12

**Authors:** Hüseyin Abacı, Ahmet Çağdaş Seçkin

**Affiliations:** Computer Engineering Department, Engineering Faculty, Adnan Menderes University, 09100 Aydın, Türkiye; huseyin.abaci@adu.edu.tr

**Keywords:** explainable artificial intelligence, internet of things, machine learning, mobile robot, positioning, wireless fidelity fingerprinting

## Abstract

Wireless Fidelity (Wi-Fi) based positioning has gained popularity for accurate indoor robot positioning in indoor navigation. In daily life, it is a low-cost solution because Wi-Fi infrastructure is already installed in many indoor areas. In addition, unlike the Global Navigation Satellite System (GNSS), Wi-Fi is more suitable for use indoors because signal blocking, attenuation, and reflection restrictions create a unique pattern in places with many Wi-Fi transmitters, and more precise positioning can be performed than GNSS. This paper proposes a machine learning-based method for Wi-Fi-enabled robot positioning in indoor environments. The contributions of this research include comprehensive 3D position estimation, utilization of existing Wi-Fi infrastructure, and a carefully collected dataset for evaluation. The results indicate that the AdaBoost algorithm attains a notable level of accuracy, utilizing the dBm signal strengths from Wi-Fi access points distributed throughout a four-floor building. The mean average error (MAE) values obtained in three axes with the Adaptive Boosting algorithm are 0.044 on the *x*-axis, 0.063 on the *y*-axis, and 0.003 m on the *z*-axis, respectively. In this study, the importance of various Wi-Fi access points was examined with explainable artificial intelligence methods, and the positioning performances obtained by using data from a smaller number of access points were examined. As a result, even when positioning was conducted with only seven selected Wi-Fi access points, the MAE value was found to be 0.811 for the *x*-axis, 0.492 for the *y*-axis, and 0.134 for the Z-axis, respectively.

## 1. Introduction

The fundamental positioning methods refer to the techniques employed to determine the location of an object with respect to a reference point. These methods find widespread application not only in various domains but extensively in navigation, measurement, and mapping. The process of determining the location using sensors is classified into relative and absolute positioning [1,2,3,4]. Relative positioning calculates the robot’s position relative to a reference point. This method utilizes a reference point or an initial position to compute the current location based on the robot’s movements and rotations. The advantages of relative positioning methods include their independence from maps or reference points and their cost-effectiveness. Sensors for relative positioning, such as encoders, gyroscopes, and speed sensors, can calculate the robot’s position directly from information related to its movements. These methods are typically in real-time and possess rapid responsiveness. However, relative positioning methods may accumulate errors over time and have limitations in accuracy. Additionally, determining the accurate initial position of the robot is crucial. By contrast, absolute positioning involves the robot determining its precise location based on external references, such as maps, landmarks, or sensors. This method employs a pre-mapped environment or detected markers through sensors to establish the robot’s location. The advantages of absolute positioning methods include providing more accurate and reliable location information, often relying on mapping or markers. Absolute positioning sensors like LiDAR, cameras, or GNSS can determine the robot’s exact position based on external references. These methods may exhibit less tendency to accumulate errors and possess higher accuracy. However, absolute positioning methods might require additional equipment or infrastructure in certain cases, making them costlier. Additionally, in some environments, the absence of markers or reference points can pose challenges.

Simultaneous localization and mapping (SLAM) is the most frequently used method in robotic indoor applications. SLAM is an algorithm set utilized to concurrently create environmental maps for mobile robots and determine their positions [5,6,7,8]. The popular sensors used for indoor SLAM include LiDAR [9,10,11,12], cameras [4,5,8,13], Wi-Fi [3,14,15], and Ultra-Wideband (UWB) positioning systems [16,17]. LiDAR SLAM is suitable for numerous applications due to its precision, real-time data processing, low error rate, and adaptability to diverse environments. Nonetheless, challenges arise in closed environments, such as mapping issues, due to factors like high cost, low resolution, and patterns formed in reflections or repetitive/uniform structures [18,19]. Given their affordability, camera systems are employed in Visual-SLAM studies across a wide range of applications, including aerial vehicles [20,21,22,23], wheeled or legged mobile robots [24,25,26], and smart vehicles [27,28]. However, challenges, like reduced performance in low light conditions or reflective surfaces, higher error rates, and data processing difficulties, can occur [5,27,29]. UWB positioning employs ultra-wideband signals to determine the locations of objects or mobile robots, providing precise location data using short-duration, high-bandwidth pulse signals. UWB technology offers millimeter-level precision [30,31]. UWB signals can penetrate walls, obstacles, or other background noise, making UWB positioning systems suitable for indoor or densely populated environmental conditions. However, installation and infrastructure requirements for UWB positioning systems can be complex and costly for wide-ranging applications [30,31,32].

Wi-Fi fingerprint-based indoor positioning is a method of using wireless Wi-Fi signals to determine the location of a device indoors [3,33,34]. This method allows mobile devices to determine their location by detecting surrounding Wi-Fi access points and evaluating the received signal strength indicator (RSSI), service set identifier (SSID), basic service set identifier (BSSID), MAC address, and other characteristics of these points. Wi-Fi can be used for fingerprint-based indoor positioning, indoor navigation, customer analytics in retail stores, routing in conference centers, and many other applications. The use of Wi-Fi fingerprinting in mobile robot positioning offers advantages, such as low computing cost, simple deployment, and the potential for multi-sensor fusion to enhance accuracy and stability [35,36,37,38]. While RSSI has been traditionally used, the introduction of round trip time has provided a more consistent and accurate alternative for positioning, especially in scenarios with few access points [39]. The fusion of Wi-Fi based positioning with other sensors like radio-frequency identification (RFID) [40], Bluetooth, ZigBee [41,42], and inertial measurement unit (IMU) [37] data can help increase robustness and accuracy in indoor environments.

Explainable artificial intelligence (XAI) refers to the ability to make understandable and expressible the logic behind the decisions made by machine learning models [43,44]. Consequently, users, developers, and system operators can gain a better understanding of why specific decisions are made by artificial intelligence systems, fostering trust. The utilization of XAI in indoor positioning systems encompasses elucidating decision-making processes, determining the importance ranking of features, monitoring system performance, ensuring privacy and security, guiding optimization strategies, and providing user education. Studies employing this approach have demonstrated the potential to provide system operators and users with a more reliable, transparent, and comprehensible indoor positioning experience [45,46].

In current studies, positioning has been achieved utilizing various existing infrastructures. Given that Wi-Fi infrastructures are readily available in these studies, all devices have been utilized, resulting in high performance when all are operational [15,34,41,42,47,48,49,50]. In all these studies, the number of Wi-Fi access points exceeds the minimum requirements for positioning applications. This study investigates datasets where Wi-Fi infrastructure achieves optimal performance by analyzing crucial Wi-Fi access points, enabling localization under diverse conditions. Thus, it aims to identify which Wi-Fi access points can be maintained for continued localization in scenarios involving potential attacks, disasters, or infrastructure changes, at least in terms of minimizing data usage for localization purposes. In this paper, we propose a system that utilizes the Wi-Fi infrastructure, derived from address and radiation patterns, as an absolute positioning system for a wheeled mobile robot in indoor environments. The secondary aim is to identify which access points are crucial and to seek answers to how the optimal number of access points with high performance can be achieved. Wi-Fi fingerprinting studies particularly aim at indoor localization based on mobile phones [15,34,47,48,49,50]. In these studies, collecting data with various smartphone brands is essential. Such endeavors can pose challenges to personal data security and privacy. This study specifically targets the utilization of hardware, namely Raspberry Pi 4, commonly employed in robotic and IoT research. The selection of this device is primarily due to its status as one of the most popular single-board computers. Unlike other single-board computers, such as Jetson Nano and BeagleBone, Raspberry Pi 4 is chosen for its integrated standard wireless module. The contributions aimed to be achieved through this work can be summarized as follows:

- Creation of a new Wi-Fi fingerprint dataset using Wi-Fi’s built-in hardware of a widely-used single-board computer, Raspberry Pi, for the purpose of utilizing address, channel, and signal strength.
- Implementation of the localization process in the dataset using machine learning and explainable AI techniques.
- Selection of transmitters for spatial analysis and planning purposes.

Detailed sections in subsequent parts of this paper outline the materials used, data collection methodologies, dataset structure, machine learning outcomes, explainable AI insights, discussion, and conclusions, providing a comprehensive overview of the contributions of this study.

## 2. Materials and Methods

**Figure 1:**
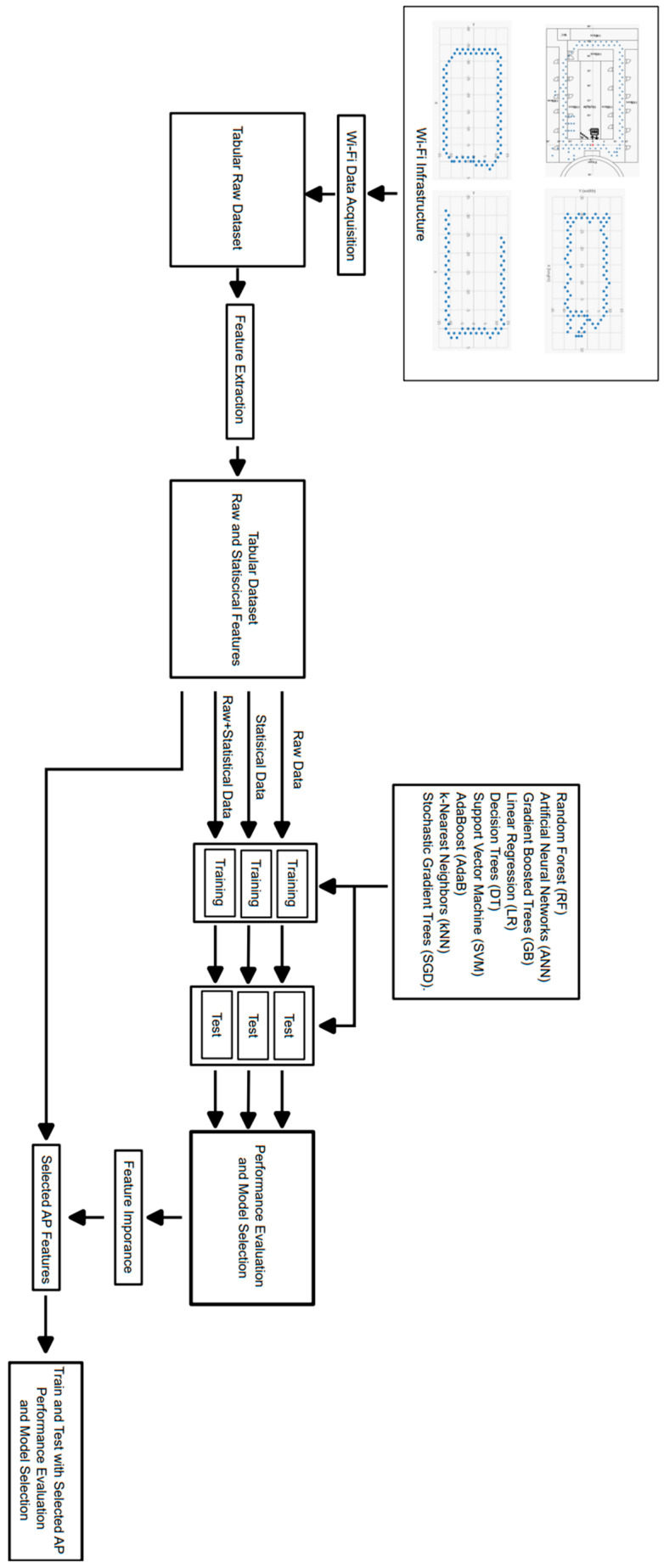
General Structure of proposed method.

The proposed methodology is outlined in Figure 1. Initially, data was gathered from specific locations in the physical space through the utilization of a Wi-Fi logger script. The recorded data was stored in its raw tabular form. Statistical feature extraction was then applied to the raw data to generate a more generalized dataset. In this newly obtained dataset, separate training and testing were conducted using various learning algorithms on raw, statistical, and combined raw and statistical datasets. Subsequently, the best models were chosen based on the performance metrics. Employing XAI methods, specifically the feature importance technique, the most crucial access points were identified. Lastly, the model underwent retraining using the selected subset of access point features, followed by performance evaluation.

### 2.1. Data Collection Process and Dataset Properties

**Figure 2:**
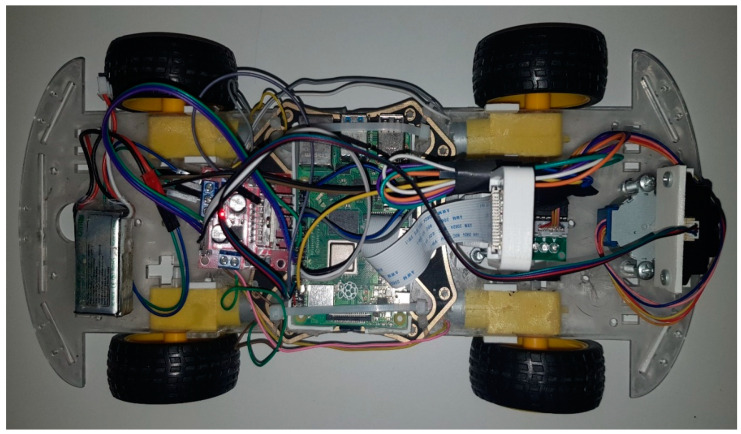
Raspberry Pi-enabled mobile robot scans the Wi-Fi signals through the building.

Raspberry Pi is a widely adopted single-board computer platform in mobile robot studies, favored by a broad user base. Its utilization in mobile robots is popular due to its capacity to fulfill various functions, such as robot control, data processing, sensor integration, and communication. The onboard Wi-Fi of Raspberry Pi is employed for communication among mobile robots. In this paper, the Wi-Fi module on Raspberry Pi is utilized for positioning through fingerprinting. Wi-Fi-based positioning using Raspberry Pi has also been explored in previous studies [51,52,53]. Raspberry Pi scans the surrounding Wi-Fi networks and determines its location using the MAC address and signal strength information of the transmitters in these networks. The fundamental standard for ground truth labeling is to use manual exact point marking. While manual methods involve laborious and time-consuming processes, instrumental methods offer more efficiency and practicality [54,55,56], because every instrument has a measurement error, and measuring with movements causes more sensor errors to accumulate. Taking individual measurements at each actual point is a more laborious method and less inaccurate than automatically collecting and labeling data with instrumental measurements. In addition, odometry methods used in indoor positioning—even if methods such as LiDAR, stereo vision, monocular vision, and sensor fusion are used—are error-cumulative and reveal serious errors over long working periods. The Raspberry Pi-enabled mobile robot determined its location by counting 30-cm tiles. It stopped and took Wi-Fi measurements each time it saw a tile line. It accomplished this by moving in a zigzag motion. At certain points, the authors manually placed the robot at specific locations. The mobile robot used for data collection purposes in this study is depicted in Figure 2.

Wi-Fi-based indoor positioning systems are effective tools that utilize wireless network signals to determine the user’s locations. However, these systems may encounter Wi-Fi-specific limitations, such as interference, multipath propagation, moving objects, and signal attenuation. Interference between Wi-Fi networks can reduce signal strength and lead to inaccurate measurements. Multipath propagation can cause signals to reach a target through multiple paths, resulting in the incorrect determination of the target’s location. Obstacles, like walls and furniture, can weaken Wi-Fi signals, making accurate positioning challenging. Living organisms or other moving objects can create a dynamic environment for positioning systems, introducing effects like absorption and reflection, leading to erroneous results. These limitations can significantly impact the accuracy and robustness of positioning systems. This article addresses the challenges faced by Wi-Fi fingerprinting-based indoor positioning systems and aims to overcome these challenges by collecting data from the same point at five different days and times.

The proposed mobile robot positioning method relies on Wi-Fi access point features, including RSSI, MAC ID, and channel features. To evaluate the effectiveness of this method, a dataset was collected within a faculty building. The data collection process involved a mobile robot equipped with a Wi-Fi adapter, camera, and LiDAR sensor. The ground truth positions of the robot were obtained from dedicated points strategically placed in the environment. Controlling the robot was achieved using a single board computer, such as Raspberry Pi, which is renowned for its cost-effectiveness and high performance in robotic applications.

**Figure 3:**
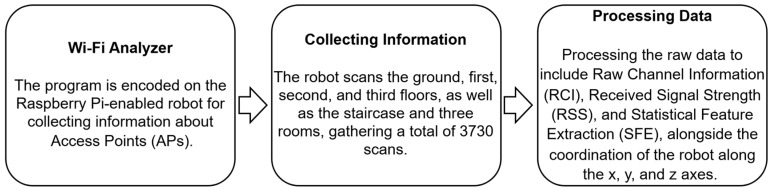
A high-level overview of the data collection process flow.

In order to gather Wi-Fi access point features, we developed a Wi-Fi analyzer program that operates on Raspberry Pi 4 (explained in Section 2.3). Specifically, we collected Wi-Fi fingerprints, including the MAC address, Wi-Fi channel ID, received signal strength in dBm, and localization information of Wi-Fi at each measurement point. The Wi-Fi analyzer recorded information from the access points (APs), repeating the process 10 times at each point. The measurement points were assigned coordinates along the x, y, and z axes, representing the length, width, and height (floors) of the building, respectively. We designated the starting point at the ground floor entrance, denoted as (x: 0, y: 0, z: 0), located at the center of the cuboid shape of Adnan Menderes University Engineering Faculty’s building. Figure 3 provides a high-level overview of the data collection process flow.

The actual locations of Wi-Fi access points are not shared because they are confidential information and within the scope of confidentiality. There are a total of 100 access points in this study. A total of 3980 scans were conducted across the 398 measurement points, capturing information from the APs. These scans encompassed the ground floor, as well as the first, second, and third floors, encompassing the entire four-floor structure of the faculty building, including stairwells and three office rooms, such as the rooms of the authors and the seminar room. The measurement points featured APs ranging from 9 to 27 and were collected with angular movement (traverse).

**Figure 4:**
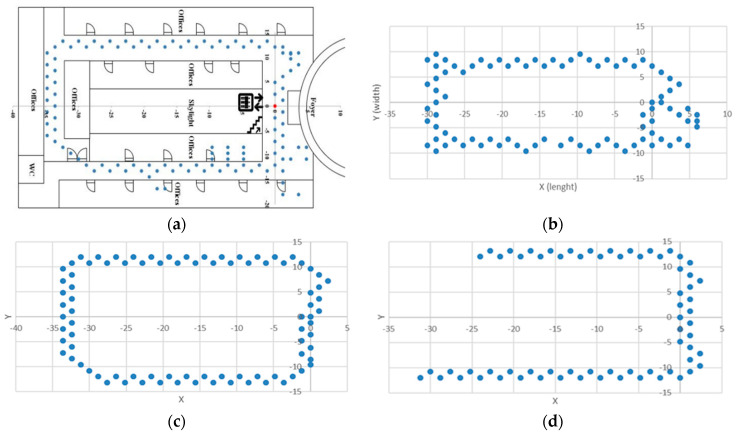
Measurement points were conducted within the Faculty Building on various floors: (**a**) Second Floor, where the Computer Engineering department is located; (**b**) Ground floor; (**c**) First floor; and (**d**) Third floor.

**Figure 5:**
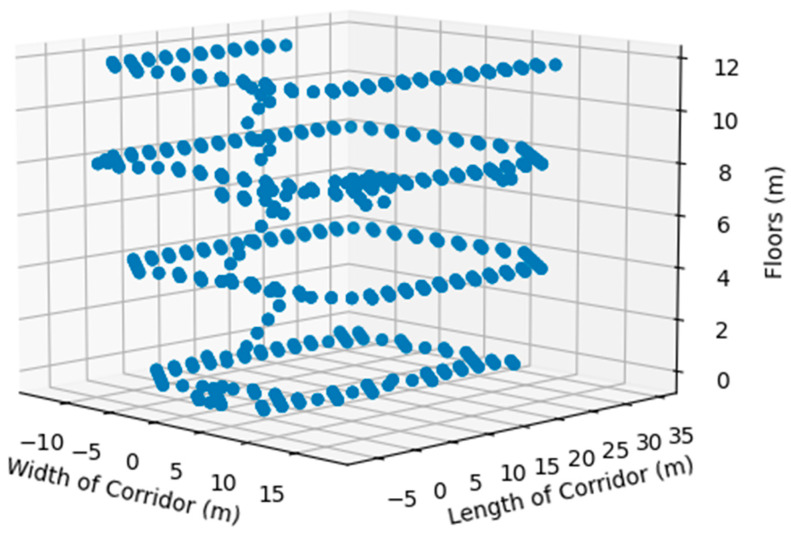
Measurement points were recorded along the x, y, and z axes.

Figure 4 illustrates the x (length) and y (width) coordinates of all Wi-Fi measurement points across the four floors, with Figure 4a highlighting the location of the Computer Engineering department on Floor 2. While the primary spatial characteristics of the floors were consistent, the layout slightly varied, and certain areas were inaccessible to our Wi-Fi analyzer-integrated robot. Figure 5 provides a comprehensive 3D plot of all the measurements taken across the floors.

At each measurement point, we collected raw channel information (RCI) of access points (Aps), including the received signal strength (RSS) in dBm, MAC numbers, and channel numbers. The collected RCIs were then subjected to statistical feature extraction (SFE), which involved extracting features, such as the maximum signal strength (in dBm), minimum signal strength (in dBm), channel transmitting the maximum and minimum signal, mean signal strength, and standard deviation of signal strength, at each measurement point. We identified the MAC addresses of all Wi-Fi signals that we scanned throughout the measurement points. Table 1 provides an overview of these extracted features.

In the given scenario, the number of Wi-Fi Aps and channels available for communication is less than the total number of Wi-Fi MAC addresses detected. This is primarily due to the presence of additional Wi-Fi signals originating from devices, such as printers, tethered mobile devices, and other Wi-Fi-enabled devices, that are not associated with an AP. Additionally, some Aps may support multiple Wi-Fi networks, further increasing the number of Wi-Fi signals detected compared to the number of Aps available. Therefore, the number of Wi-Fi Aps = the number of channels < the number of Wi-Fi MAC address.

### 2.2. Machine Learning and Training

**Figure 6:**
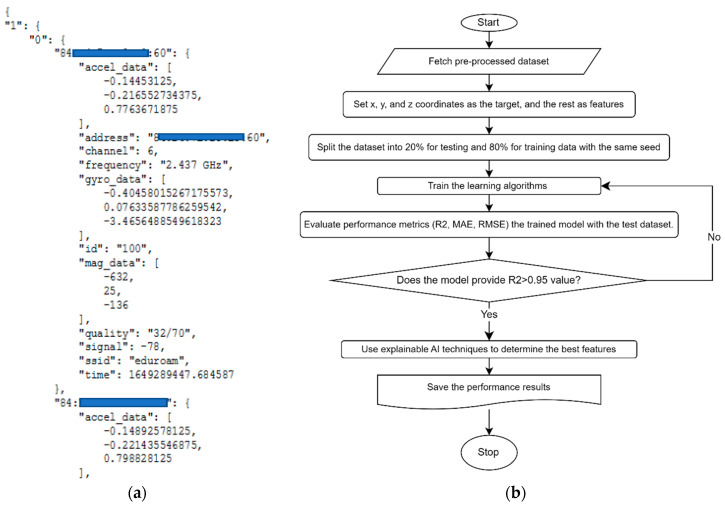
(**a**) Displays data collected at each measurement point and stored as JSON. (**b**) Illustrates the progress of the machine learning process after raw data processing.

To employ machine learning techniques, the raw data needs to be processed for ML algorithms, which can accept various types of data, including continuous and categorical variables. Figure 6a illustrates the raw data collected via Raspberry Pi and stored as a JSON object. We conducted various measurements, including channel signal quality, channel frequency, and Wi-Fi SSID. These measurements were compiled in JSON format, including the timestamp of when the Wi-Fi SSID measurement was taken, along with location information. These data were then pre-processed separately to comprise the targets and features used to train ML algorithms and test the detection of robot coordination. We split the data into 80% for training and 20% for testing purposes. Subsequently, further investigation was conducted to determine the most important features in our dataset. Figure 6b illustrates the entire machine learning process in our work, along with AI explainability steps, presented as a flow chart. First, the data is collected and subjected to data preprocessing. In the next step, the values x, y, and z are assigned as target values. After the data is split into 20% test and 80% train, it is subjected to training with various learning algorithms. Following training, performance metric evaluation is conducted. Here, performance metrics used for regression purposes are applied. This process is repeated until the R^2^ value exceeds 0.95. Then, an examination is conducted using XAI techniques, and the results are recorded, completing the process.

To predict the robot’s location, machine learning (ML) algorithms were trained using pre-collected data, followed by testing the trained model. In this study, we employed well-known ML algorithms trained on the features described in Table 1. The algorithms were trained using a combination of raw channel information (RCI), statistical feature extraction (SFE), and both RCI and SFE features. The ML algorithms utilized in this research encompass random forest (RF), artificial neural networks (ANNs), gradient boosted trees (GB), linear regression (LR), decision trees (DTs), support vector machine (SVM), AdaBoost (AdaB), k-nearest neighbors (kNNs), and stochastic gradient trees (SGD). The algorithms used in this study are briefly explained below, and scikit-learn and XGBoost libraries were employed in the use of these algorithms [57,58]. RF is an ensemble learning algorithm that consists of multiple decision trees. Each tree is trained on randomly selected subsets of data, and their aggregated predictions aim to improve overall accuracy. An ANN is a model inspired by biological neural systems, composed of interconnected artificial neurons arranged in layers. It processes input data, learns patterns, and models complex relationships. GB is another ensemble technique, combining weak learners, typically decision trees, to create a strong predictive model. It sequentially adds new trees, correcting errors made by previous ones. Logistic regression (LR) is a linear model primarily used for classification tasks. It analyzes the relationship between input features and outputs to categorize data into specific classes. A DT is a tree-like structure used for classification or regression. It splits data based on featured values, constructing a flowchart of decisions. An SVM is a supervised learning algorithm that seeks to find the optimal decision boundary with the maximum margin between different classes. AdaB is an ensemble method that also combines weak learners, with a focus on examples of how prior models performed poorly, thus improving accuracy incrementally. A kNN algorithm determines the class or value of a data point based on its proximity to neighboring points. An SGD is an optimization algorithm designed for training machine learning models on large datasets. It minimizes a specific objective function using gradient descent. The algorithms and their corresponding hyperparameters used in this study are summarized in Table 2. To ensure optimal performance of our machine learning models, we conducted an extensive iterative grid search to identify the best hyperparameters for each of the nine algorithms used in this study. Leveraging our experience in the field and systematic testing, we evaluated a range of parameter combinations, such as learning rate, max depth, and number of estimators, among others. The values presented in Table 2 were found to be the most effective configurations for the scenario of this study, achieving maximum accuracy in Wi-Fi-based indoor positioning. In all analyses conducted throughout this study, the hyperparameters in Table 2 were consistently applied to maintain robustness and comparability of the results.

Mean absolute error (MAE), root mean square error (RMSE), and the coefficient of determination (R^2^) are crucial metrics in regression analysis, each serving specific purposes and providing distinct insights into the performance of a regression model. Their applications in Wi-Fi positioning studies highlight their significance in evaluating and optimizing predictive models. MAE measures the average magnitude of errors in a set of predictions, without considering their direction. It is calculated as in Equation (1). Where yi are the actual values and ŷi are the predicted values. MAE is straightforward to interpret, providing a direct indication of the average prediction error. This metric is particularly useful when the goal is to understand the typical size of the errors made by the model. MAE is useful for providing an intuitive measure of average error, helping to understand the typical deviation from the actual location. RMSE also measures the average magnitude of the error, but it squares the differences between the predicted and actual values before averaging, which emphasizes larger errors. RMSE is presented as Equation (2). Where yi are the actual values and ŷi are the predicted values. It is more sensitive to outliers than MAE because the squaring process increases the impact of larger errors. This makes RMSE a more appropriate metric when large errors are particularly undesirable. RMSE is valuable because it penalizes larger errors more heavily, which is important in positioning where large deviations can significantly impact usability and accuracy. R^2^ indicates the proportion of the variance in the dependent variable that is predictable from the independent variables. R^2^ is calculated as Equation (3). Where yi are the actual values, ŷi are the predicted values and $\bar{y}$ is the mean of the actual values. R^2^ ranges from 0 to 1, with higher values indicating a better fit of the model to the data. R^2^ helps in assessing how well the model captures the variance in the data, ensuring that the model not only fits the data well but generalizes effectively to new, unseen data.

(1) $$MAE=\frac{1}{n}\sum_{i=1}^{n}\left|y_{i}-{\hat{y}}_{i}\right|$$

(2) $$RMSE=\sqrt{\frac{1}{n}\sum_{i=1}^{n}{\left(y_{i}-{\hat{y}}_{i}\right)}^{2}}$$

(3) $$R^{2}=1-\frac{\sum_{i=1}^{n}{\left(y_{i}-{\hat{y}}_{i}\right)}^{2}}{\sum_{i=1}^{n}{\left(y_{i}-\bar{y}\right)}^{2}}$$

### 2.3. Explainable AI and Feature Importance

Permutation feature importance (PFI) is a technique used in explainable artificial intelligence (XAI) to assess the importance of individual features in machine learning models to help understand which features or variables have the greatest impact on a model’s predictions breed [44,59,60]. This method is particularly useful for black box models, such as random forests, gradient boosting, and neural networks, when describing the internal performance of a model may not be straightforward. The idea behind PFI is to measure how model performance decreases as values a feature change randomly, effectively breaking the link between that feature and the target variable as performance decreases, the importance of the feature is assumed to increase.

The calculation process for permutation feature importance involves assessing the importance of each feature in a machine learning model. For each feature in the dataset, you randomly shuffle the values of that feature across all data points, effectively breaking the relationship between that feature and the target variable. Then, you use the model to make predictions on the modified dataset and calculate a performance metric, typically referred to as the “permutation score”. This score reflects the model’s performance when the feature’s values are permuted. By comparing the permutation score to the original performance score (i.e., the score on the unmodified dataset), you can determine the impact of each feature. A significant drop in performance suggests that the feature is important, and the features are ranked based on the magnitude of this drop to assess their relative importance.

The importance score of each component is calculated as the difference between the baseline performance score and the change score. A sharp decrease in performance results in a higher importance score for that item. The items are ranked based on their importance scores. Significant decreases in activity are considered more important.

## 3. Results and Discussion

**Figure 7:**
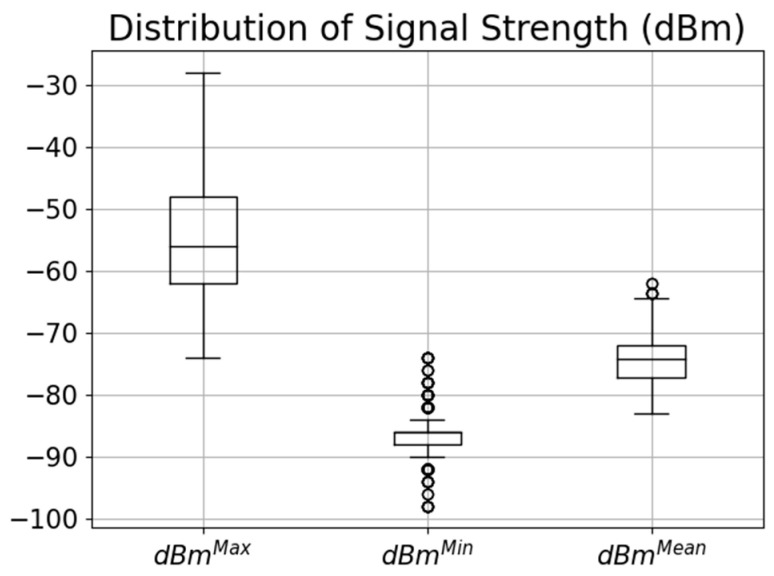
Distribution of RSS measured at all points.

The experiment aimed to assess the performance of the system in various scenarios and evaluate its capability to accurately estimate the position of the robot using Wi-Fi RSS measurements. Multiple machine learning algorithms were compared to determine the most accurate model for position prediction. Wi-Fi signals can face interference from various sources, like neighboring networks on the same channels, electronic devices, and physical obstacles. This interference often causes signal disruptions, slower speeds, and increased latency. To counter this, optimizing router placement, selecting less crowded channels, and using newer Wi-Fi standards can improve signal reliability. Understanding and addressing interference sources are vital for a stable Wi-Fi connection. Figure 7 illustrates the distribution of RSS values measured at different points, depicting the maximum, minimum, and mean values. The worst RSS measurement recorded was −98 dBm, indicating a weaker signal strength, while the best measurement was −27 dBm, representing a stronger signal strength. These measurements provide insights into the range and variability of RSS values observed during the experiment, highlighting the varying signal strengths experienced at different points in the environment.

### 3.1. Model and Feature Selection

Multi-objective regression or classification refers to the task of simultaneously predicting multiple targets or objectives using machine learning models. In the context of mobile indoor positioning based on Wi-Fi RSS, multi-objective regression is employed to predict the *x*-axis (representing the length of the building), *y*-axis (representing the width of the building), and *z*-axis (floor—representing the height of the building) coordinates for precise positioning. In this study, the machine learning algorithms listed in Table 2 are trained sequentially to predict each axis independently. Initially, the algorithms are trained to predict the *x*-axis coordinate using RCI and SFE features. Subsequently, the algorithms are trained to predict the *y*-axis coordinate. Finally, the algorithms are trained to predict the *z*-axis coordinate, incorporating the data as well as the previously predicted x and *y*-axis coordinates. Table 3 presents the performance results (RMSE, MAE, and R^2^) of the algorithms for the *x*-axis using RCI, SFE, and their combination as training features. The best-performing values for each performance metric are highlighted in bold. AdaB consistently demonstrates the best performance across all cases. The experimental findings indicate that multi-objective regression enables accurate positioning in mobile indoor environments, with AdaB exhibiting superior performance in predicting the *x*-axis coordinate based on Wi-Fi RSS measurements.

**Figure 8:**
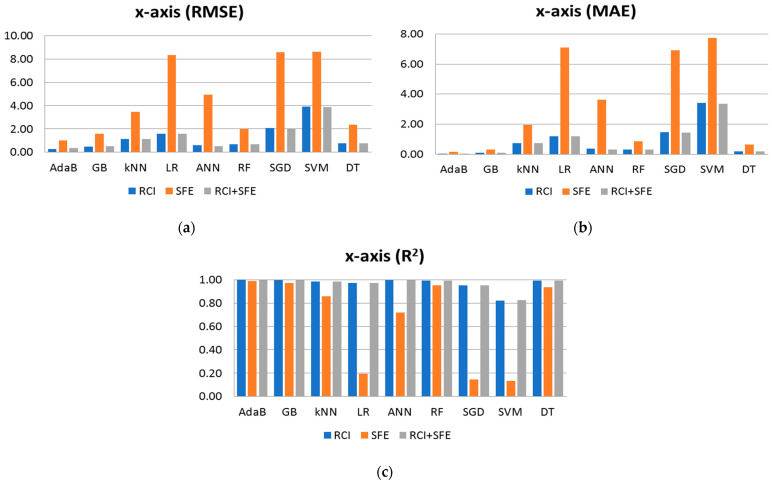
Shows performance result of algorithms when predicting the x axis using RCI, SFE and RCI + SFE features: (**a**) Performance results of RMSE; (**b**) Performance results of MAE; (**c**) Performance results of R^2^.

The results depicted in Figure 8 showcase the performance metrics of the algorithms individually, presented in bar charts. It is evident that when used in isolation, the SFE features yield the least favorable results. However, when combined with RCI, a notable enhancement in performance is observed. After conducting a thorough analysis of all the results, it becomes evident that both RCI and SFE demonstrate accuracy in predicting the robot’s position along the *x*-axis. While RCI alone provides similar performance results, using all features together generally yields improved results across almost all algorithms. The exception is AdaB, which achieves slightly better results when using RCI alone. Notably, while the SFE features exhibit comparatively lower performance, the observed error margins remain within an acceptable range for a wide range of real-world applications. For instance, SFE used for AdaB achieves an MAE of approximately 0.156 m (approximately 15 cm) in positional error, which is deemed acceptable given the context of the problem at hand. Furthermore, the figures illustrate those algorithms, such as AdaB, GB, DT, and ANN, perform admirably, with AdaB achieving the lowest MAE in Figure 8b at 0.0445 m, akin to a centimeter-scale error. This centimeter-scale accuracy is considered highly suitable for indoor positioning applications.

**Figure 9:**
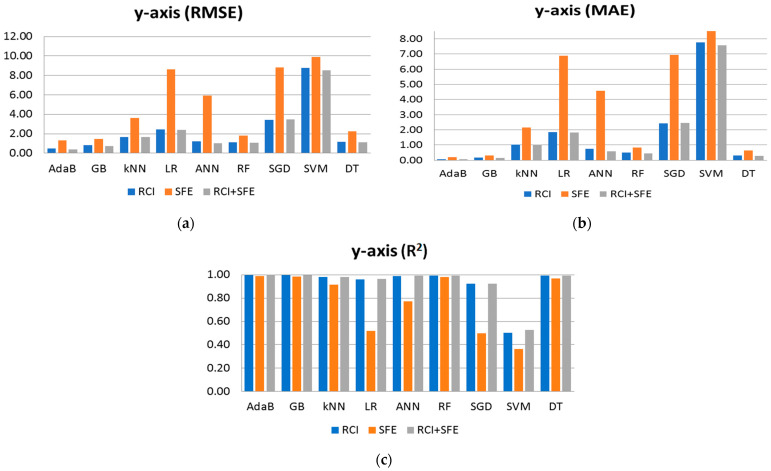
Shows performance result of algorithms when predicting the y axis using RCI, SFE and RCI + SFE features: (**a**) Performance results of RMSE; (**b**) Performance results of MAE; (**c**) Performance results of R^2^.

**Figure 10:**
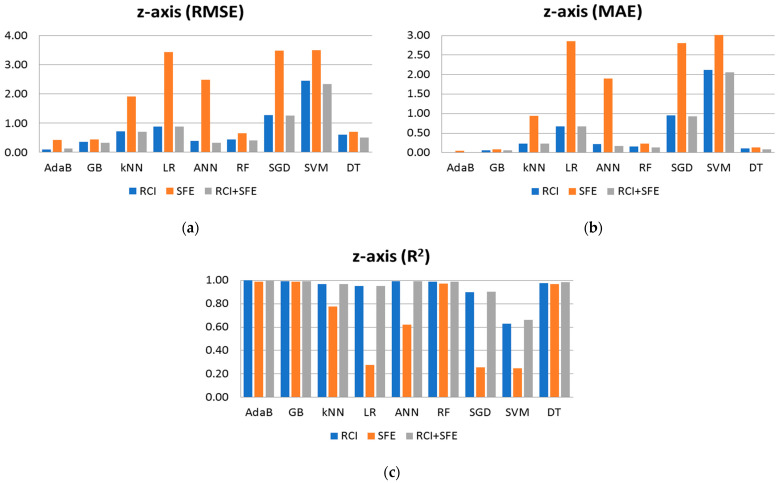
Shows performance result of algorithms when predicting the z axis using RCI, SFE and RCI + SFE features: (**a**) Performance results of RMSE; (**b**) Performance results of MAE; (**c**) Performance results of R^2^.

Table 4 presents the performance results, including RMSE, MAE, and R^2^, for the algorithms applied to the *y*-axis. These results are obtained using RCI, SFE, and their combined features for training. Notably, AdaB consistently demonstrates superior performance across all cases. While the results for the *y*-axis exhibit a similar trend to those of the *x*-axis, slight reductions in accuracy are observed. It is essential to highlight that predicting the *y*-axis poses unique challenges, primarily due to obstructions present along the path of Wi-Fi access points aligned with the *y*-axis. Accurate estimation necessitates consideration of multiple access points situated within the same corridor, each at a different position along the *y*-axis. Figure 9 presents the results of Table 4 in bar charts, analyzing the performance results separately. AdaB and GB perform well on the *y*-axis, while LR, SVM, and SGD yield the worst results, as observed in the *x*-axis analysis. Approximately centimeter-scale errors are also observed for the *y*-axis. The R^2^ results are close to 1 (0.999), indicating successful model performance considering the scale of the target values. Table 5 showcases the performance results (RMSE, MAE, and R^2^) of the algorithms for the *z*-axis. Our observations suggest that estimating the floor of the robot is easier compared to the other axes, including stairwells where the robot’s height falls between floors. Figure 10 illustrates the outcomes of Table 5 through bar charts. The performance results prominently indicate that AdaB, GB, and DT exhibit superior performance. Notably, AdaB achieves the highest MAE accuracy, operating within a millimeter-scale error margin.

### 3.2. Explaining the Selected Model

The previous section examined the well-performing algorithms and feature sets. In order to explain these outcomes, we investigated the top three individual features that yielded the best results for the x, y, and z axes separately. The earlier results demonstrated the favorable performance of the AdaB algorithm when utilizing the RCI feature set. Furthermore, these results highlight that when combining RCI with SFE, the performance closely aligns with that achieved by using RCI alone, especially with AdaB, and notably, it delivers improved performance for other algorithms. AdaB trains weak learners (usually referred to as decision trees) sequentially, focusing on the errors of the previous model. Each weak learner attempts to perform better on the data points that were more heavily weighted due to the errors of the previous learner. Ultimately, these weak learners are combined to create a stronger model.

**Figure 11:**
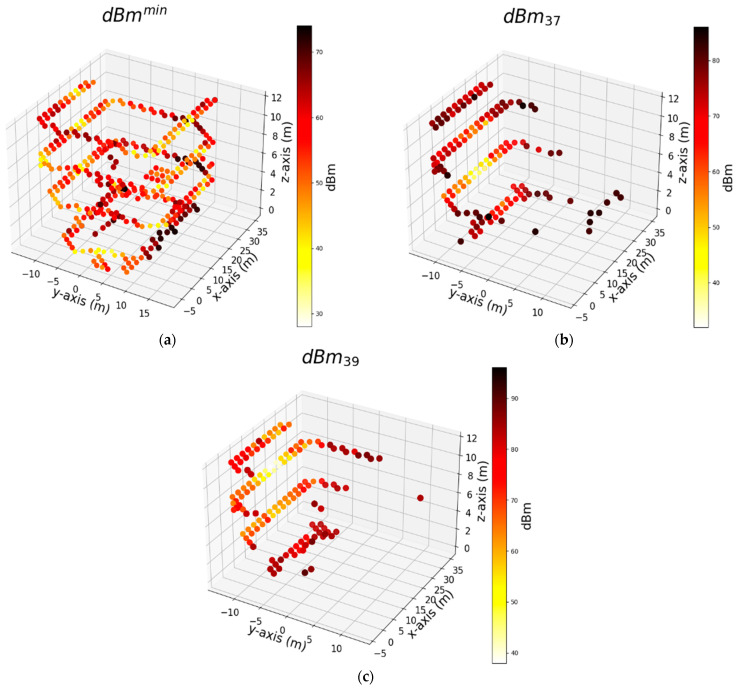
Shows top 3 features used by the algorithm that successfully predicts measurement points on the length of corridors (*x*-axis) respectively: (**a**) Minimum signal strength (dBm) of Wi-Fi within whole dataset at the measurement points; (**b**) Signal strength (dBm) of Wi-Fi that numbered as 37; (**c**) Signal strength (dBm) of Wi-Fi that numbered as 39.

**Figure 12:**
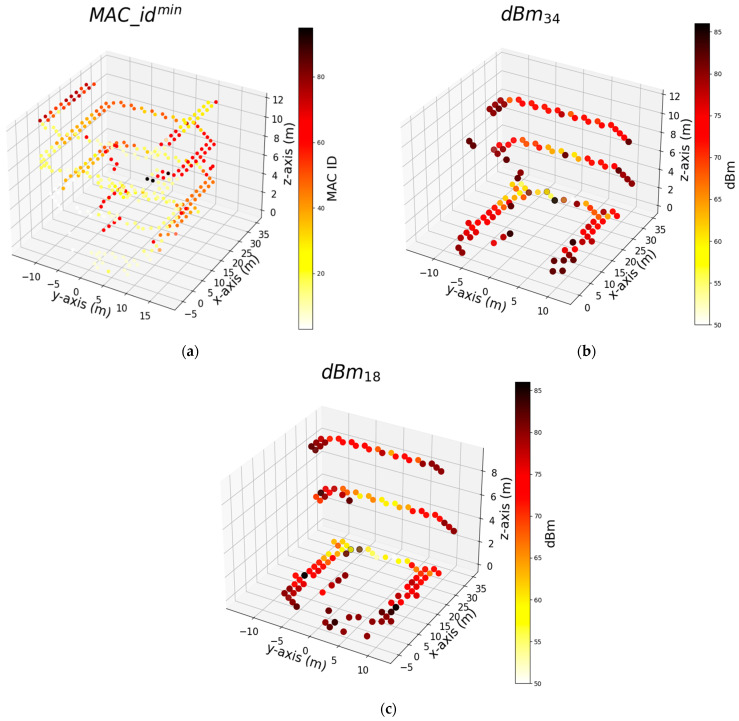
Shows top 3 features used by the algorithm that successfully predicts measurement points on the width of corridors (*y*-axis) respectively: (**a**) ID of Wi-Fi has minimum signal strength (dBm) at each measurement points; (**b**) Signal strength (dBm) of Wi-Fi that numbered as 34; (**c**) Signal strength (dBm) of Wi-Fi that numbered as 18.

**Figure 13:**
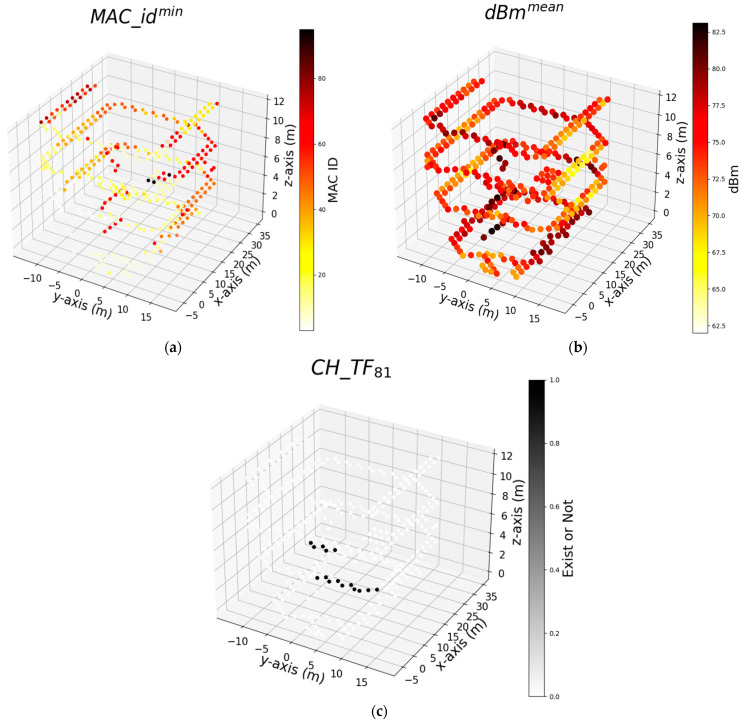
Shows top 3 features used by the algorithm that successfully predicts measurement points on the floor of the building (*z*-axis) respectively: (**a**) ID of Wi-Fi has minimum signal strength (dBm) at each measurement points; (**b**) Average signal strength (dBm) of all Wi-Fi at the measurement points; (**c**) Signal of the MAC ID 81 exist or not at the measurement points.

To gain a more comprehensive understanding of the feature set dynamics, we proceeded to explore the combined use of RCI and SFE feature sets. Thus, Figure 11, Figure 12 and Figure 13 illustrate the top three features of the AdaB model utilizing the RCI + SFE features. Figure 10a displays the minimum dBm values at the measurement points, represented as ${dBm}^{min}$, which had the most significant impact on the results of the AdaB algorithm. The varying shades of yellow indicate a decrease in the dBm at measurement points, while darker colors indicate an increase. This feature spans across the building, aiding in the accurate positioning of the robot along the *x*-axis. This individual feature is followed by the dBm values of the Wi-Fi signals identified as ${dBm}_{37}$, and subsequently by ${dBm}_{39}$.

In Figure 11b, the second influential feature for the AdaB model, which is ${dBm}_{37}$, is depicted. The RSS of the corresponding access point (AP) exhibits strong signal strength in the left corridor of the first floor, represented by a yellow color. The power of ${dBm}_{37}$ weakens as we move towards the second, third, and ground floor. Moreover, the signal strength of ${dBm}_{37}$ diminishes in the right corridor across the different floors. A similar trend was observed for the feature ${dBm}_{39}$. It is important to note that the model’s ability to determine its position is influenced not only by the strength of the RSS signals but by the weakness of the RSS signals.

Figure 12 illustrates the top three features utilized by the algorithm that successfully predicts the measurement points along the width of the corridors (*y*-axis). The MAC ID, denoted as ${MAC\text{\_}id}^{min}$, which represents the minimum signal strength at the measurement point, has the most significant contribution to the AdaB model. This is followed by ${dBm}_{34}$, the RSS influences the determination of positioning on the *y*-axis.

Figure 13 displays the three most effective features for predicting the *z*-axis. The floor height of the robot can be easily determined by examining the ${MAC\text{\_}id}^{min}$, ${dBm}^{mean}$, which is the average of all measured signals at the points and ${CH\text{\_}TF}_{81}$, whether the signal strength of the MAC ID 81 exists or not at the measurement points. Notably, the feature ${MAC\text{\_}id}^{min}$ proves to be an influential feature for both the *y*-axis and *z*-axis.

### 3.3. Selecting Minimum Number of Wi-Fi AP

**Figure 14:**
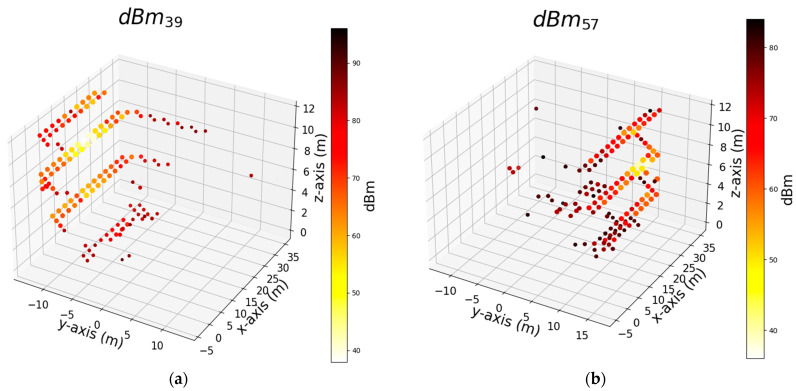
Shows top 2 signal strength (dBm) of Wi-Fi that were used by the AdaB algorithm to successfully predict measurement points on the length of corridors (*x*-axis) respectively: (**a**) Rank 1 feature (Wi-Fi) that numbered as 39; (**b**) Rank 2 feature (Wi-Fi) that numbered as 57.

**Figure 15:**
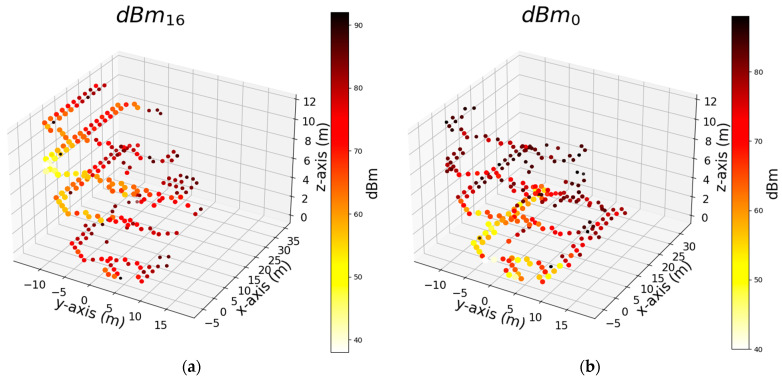
Shows top 2 signal strength (dBm) of Wi-Fi that were used by the AdaB algorithm to successfully predict measurement points on the width of corridors (*y*-axis) respectively: (**a**) Rank 1 feature (Wi-Fi) that numbered as 16; (**b**) Rank 2 feature (Wi-Fi) that numbered as 0.

**Figure 16:**
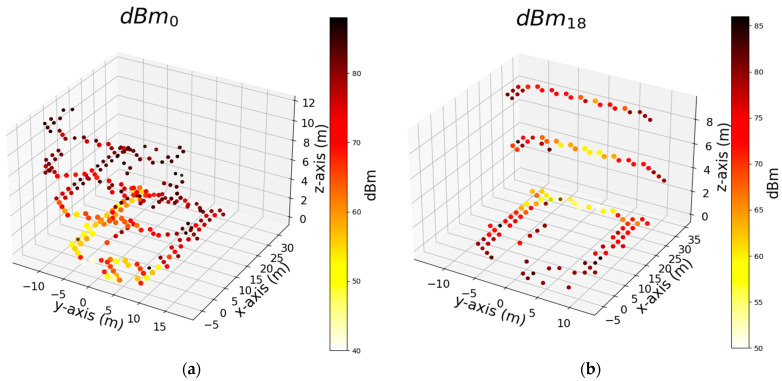
Shows top 2 signal strength (dBm) of Wi-Fi that were used by the AdaB algorithm to successfully predict measurement points on the floor of the building (*z*-axis) respectively: (**a**) Rank 1 feature (Wi-Fi) that numbered as 0; (**b**) Rank 2 feature (Wi-Fi) that numbered as 18.

We trained the algorithms using three sets of features, and the results are presented above. Among the algorithms considered, AdaB demonstrated the best performance overall. In this section, we focus on the model trained solely with the dBm feature of the Wi-Fi signal to simplify the feature set and determine its effectiveness in robot positioning. Thus, we delve deeper into the RCI feature set to identify the most influential and effective Wi-Fi Aps within the building. Our goal is to pinpoint specific Aps that can offer performance similar to using the entire set of Wi-Fi Aps. To perform this, we start by identifying the individual features that yield the best results for the x, y, and z axes independently. Then, we compare how the performance changes when we add each of these top features (dBm of Wi-Fi) one by one. Our aim is to find the point at which adding more features no longer significantly improves performance, effectively achieving the same level of performance with a minimal set of features. Table 6 shows the top seven features that exhibit the most significant impact on the AdaB algorithm’s performance, evaluated using the MAE and R^2^ metrics for the x, y, and z coordinates. For this purpose, we employed the R^2^ and MAE performance metrics and utilized the permutation feature importance (PFI) technique with a permutation count of five to assess the feature significance within the machine learning model. PFI involves the systematic shuffling of each feature’s values to measure the resulting impact on the model’s performance. This aids in feature selection and model interpretability, even though its iterative evaluation process can be computationally intensive. Figure 14, Figure 15 and Figure 16 depict the distribution of the top two Wi-Fi dBm features in a 3D plot. The measurements close to these Wi-Fi signals exhibit signal strengths ranging from −38 to −40 dBm.

**Figure 17:**
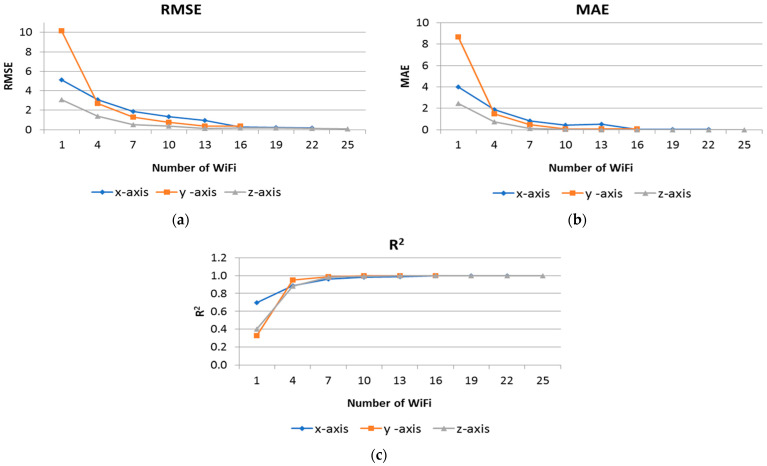
Saturation points of the RMSE, MAE, and R^2^ performance metrics when utilizing 1, 4, 7, and up to 25 of the most significant features for AdaB in the x, y, and z axes. (**a**) RMSE of most significant features; (**b**) MAE of most significant features; (**c**) RMSE of most significant features.

Furthermore, we assessed the adequacy of available AP signals. Figure 17 illustrates the effect of progressively increasing the number of Wi-Fi dBm signals on performance metrics, including RMSE, MAE, and R^2^. These figures indicate that a minimum of seven dBm signals is adequate for achieving robot positioning with less than 1 m error for some situations. However, for highly accurate positioning, MAE results suggest that 16 Wi-Fi signals are necessary for the *x*-axis, 10 for the *y*-axis, and 7 for the *z*-axis. Furthermore, the figures indicate that for applications where precision positioning is not critical, four Wi-Fi signals with an average error margin of approximately 1 to 2 m are also sufficient.

Table 7 presents the performance results of AdaB when employing various feature sets, including using all dBm signals, the RCI feature set, utilizing the most effective seven Wi-Fi signals, and the most effective 10 signals. The distinctions between first two feature sets are minimal and negligible. Given the additional overhead involved in collecting, processing, and training with a larger feature set, opting for a smaller feature set would provide accurate results with greater efficiency.

**Figure 18:**
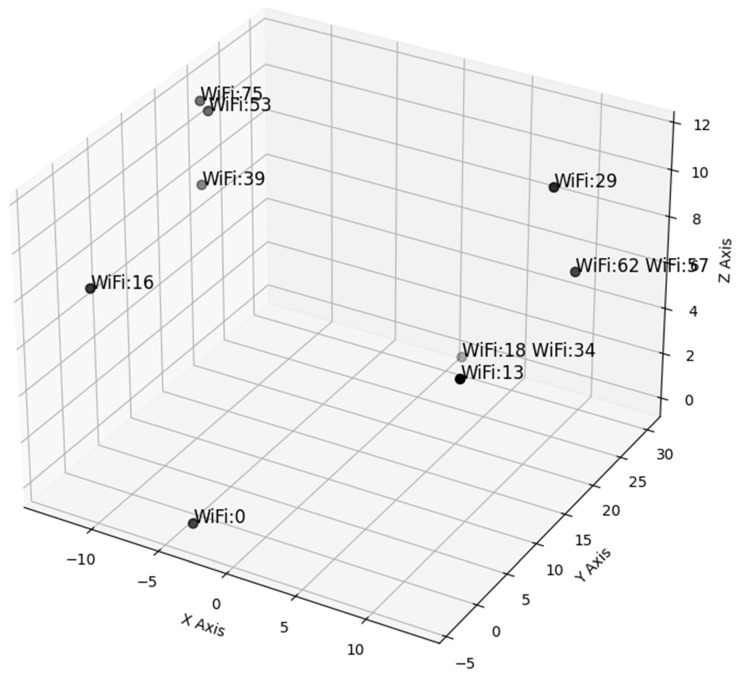
Locations of the most significant 11 Wi-Fi signals.

Utilizing only the dBm signals from Wi-Fi IDs 39, 57, 53, 62, 16, 0, 34, 13, 18, 75, and 29 yields a satisfactory MAE (mean absolute error). Figure 18 depicts the positions of the 11 Wi-Fi points used to derive the values in Table 8, covering the x, y, and z coordinates. Furthermore, employing Wi-Fi points 0, 16, 18, 34, 39, 53, 57, and 75, selected as the top four ranked Wi-Fi points from Table 6, results in an MAE ranging between 0.5 and 1.27. However, when all Wi-Fi points shown in Figure 18 are used, the MAE decreases to less than 0.17 across all axes, corresponding to an error margin of approximately 17 cm.

### 3.4. Comparison of Proposed Method with Existing Systems

In this study, we developed and evaluated a method for Wi-Fi-based indoor positioning using machine learning, supported by a newly collected dataset tailored to this purpose. The dataset, gathered using a single-board Raspberry Pi 4, consists of signal strength measurements from 398 unique locations within a 3500 m^2^ indoor area, utilizing 100 access points (APs). Unlike many existing studies that incorporate additional technologies, such as ZigBee or Bluetooth, to enhance accuracy, our approach relies solely on Wi-Fi signals, offering a cost-effective and practical alternative for a variety of applications.

The proposed model achieves notable precision in three-dimensional (3D) localization, with mean absolute error (MAE) values of 0.044 m on the *x*-axis, 0.063 m on the *y*-axis, and 0.003 m on the *z*-axis. Using the Euclidean distance formula, the total 3D positioning MAE is calculated as follows:

(4) $${MAE}_{3D}=\sqrt{{\left({MAE}_{x}\right)}^{2}+{\left({MAE}_{y}\right)}^{2}+{\left({MAE}_{z}\right)}^{2}}$$

Substituting the values, the overall positioning error is approximately 0.077 m. This level of accuracy highlights the effectiveness of Wi-Fi fingerprinting for indoor localization without relying on additional assistive technologies.

Table 9 presents a comparative analysis of our method against existing Wi-Fi fingerprinting studies, considering metrics such as sample count, number of locations, access points, and device type. Our dataset offers dense sampling, with a high number of measurements per location, allowing the model to better capture variations in Wi-Fi signal strength. This sampling approach contributes significantly to the accuracy of the proposed model, particularly in large and complex indoor environments. The implementation uses a Raspberry Pi 4 device, demonstrating that accurate Wi-Fi-based positioning can be achieved with minimal infrastructure. This setup is not only cost-effective but scalable for a range of indoor applications, including robotics and smart building technologies. Compared to existing studies, our method demonstrates strong performance. For example, Reference [47] reports a 3D MAE of 6.05 m, while Reference [15] reports a 2D MAE of 2.3 m. By contrast, our model achieves sub-meter accuracy with a 3D MAE of 0.077 m. This improvement underscores the potential of machine learning techniques in addressing challenges posed by indoor environments.

By selecting the seven most influential APs, the proposed approach maintains accuracy while reducing computational complexity. The optimization of hyperparameters across nine machine learning models further enhances the model’s robustness and adaptability to the unique characteristics of the dataset. This approach provides a practical solution for environments where accurate and reliable indoor positioning is needed. By leveraging existing Wi-Fi infrastructure, the method offers a scalable and accessible alternative to more complex or costly systems. While further improvements may be explored, the results demonstrate that this method is well-suited for modern indoor environments.

Commercial Wi-Fi-based systems, such as Cisco DNA Spaces, Ekahau, Inpixion, Navigine, and Infsoft, leverage existing Wi-Fi AP infrastructure to provide indoor positioning. These systems typically utilize fingerprinting or signal triangulation methods to estimate device locations. These commercial brands state that they perform Wi-Fi based positioning and Wi-Fi planning, but no company shares information about the accuracy of these systems.

## 4. Conclusions

This study presents a comprehensive system for the global absolute positioning of a wheeled mobile robot using Wi-Fi infrastructure and leveraging the integrated wireless capability of Raspberry Pi 4. The primary contributions of this study are diverse. Firstly, it demonstrates the practicality and ease of implementation in real-world scenarios by using the hardware of the widely used single-board computer, Raspberry Pi, to create a new Wi-Fi fingerprinting data setup. Secondly, it enhances the transparency and state of the system through the application of explainable artificial intelligence (XAI) methods in the machine configuration and supervised data setup of the localization process. Thirdly, it highlights the expansion of transmitter selection for spatial analysis and planning purposes, which is crucial for optimizing spatial analyses.

The experimental results show that multi-objective regression techniques allow for precise indoor recordings, and that AdaBoost exhibits superior performance in predicting coordinates based on Wi-Fi RSS measurements. XAI techniques provide more information about feature importance, enabling users and developers to create a more understandable and reliable system. The results indicate that the combination of RCI and SFE features ensures optimal overall performance across all algorithms. The AdaB algorithm, when using only RCI, yields slightly better results with an average absolute error of 0.077 m. However, acknowledging potential variations in real-world scenarios underlines the need for ongoing research to explore the model’s adaptability. Additionally, the examination of feature sets and simplification of data collection processes have revealed excellent performance results in our four-story building using only dBm signals. Achieving near-optimal results for the *z*-axis with signals from only seven Wi-Fi access points is noteworthy. While these findings are not universally applicable, they emphasize the importance of tailoring setups to specific deployment environments.

The proposed methodology within the system demonstrates high-performance localization through the use of numerous access points, but it is crucial to accept and address the inherent limitations for broader applicability. Future research efforts should focus on refining the model to ensure robustness in diverse and dynamic indoor environments. This method not only contributes to robotic applications but plays a significant role in improving inventory management in indoor settings, creating a broader impact beyond robotics alone. Additionally, the integration of extra sensors could be explored. Furthermore, addressing issues of interference and signal variability in various indoor environments remains a critical area for ongoing research. These summarized findings and algorithms provide a solid foundation for technological advancements in indoor settings, particularly for mobile robotics and IoT applications.

## Figures and Tables

**Table 1:** Targets, features, and their explanation that are used to train algorithms.

Notation	Dataset Abbreviation	Type	Explanation
*x*-axis	tx	Target	Robot coordination on *x*-axis
*y*-axis	ty	Target	Robot coordination on *y*-axis
*z*-axis	tz	Target	Robot coordination on *z*-axis
${dBm}^{min}$	F_dB_min	Feature (SFE)	Minimum RSS value at a measurement point
${dBm}^{max}$	F_dB_max	Feature (SFE)	Maximum RSS value at a measurement point
${dBm}^{mean}$	F_dB_mean	Feature (SFE)	Mean RSS values at a measurement point
${dBm}^{std}$	F_dB_std	Feature (SFE)	Standard deviation of RSS values at a measurement point
${MAC\text{\_}id}^{min}$	F_dB_MAC_ID_min	Feature (SFE)	ID of the MAC with the minimum measured RSS
${MAC\text{\_}id}^{max}$	F_dB_MAC_ID_max	Feature (SFE)	ID of the MAC with the maximum measured RSS
${AP\text{\_}CH}^{min}$	F_dB_CH_min	Feature (SFE)	Channel of the AP with the minimum measured RSS
${AP\text{\_}CH}^{max}$	F_dB_CH_max	Feature (SFE)	Channel of the AP with the maximum measured RSS
${CH}^{uniq}$	F_nofCH	Feature (SFE)	Number of unique channels in a measurement point
${MAC}^{count}$	F_nofMAC_ID	Feature (SFE)	Number of MAC IDs in a measurement point
${CH\text{\_}Freq}^{most}$	F_CH_maxfrequent	Feature (SFE)	Most frequent Channel number in a measurement point
${CH\text{\_}Freq}^{least}$	F_CH_minfrequent	Feature (SFE)	Least frequent Channel number in a measurement point
${CH\text{\_}TF}_{x}$	MAC_ID_X	Feature (RCI)	Whether MAC ID-X [0 to 99] exists at a measurement point or not.
${CH}_{X}$	CH_X	Feature (RCI)	Channel number of MAC ID-X [0 to 99] (if doesn’t exist set to 0)
${dBm}_{x}$	dBm_X	Feature (RCI)	RSS of MAC ID-X [0 to 99] (if doesn’t exist set to 0)

**Table 2:** The employed algorithms and their corresponding hyperparameters.

Algorithm	Hyperparameters
RF	Number of trees (10), Max number of features (unlimited), Replicable training (No), Max depth (unlimited), Stop splitting subsets (5)
ANN	2 Hidden layers (1st layer 100 neurons, 2nd layer 20 neuron), Activation Function: ReLu, Solver for weight optimization: Adam, Strength of the L2 regularization term (Alpha): 0.0001, Maximum number of iterations: 200, The initial learning rate: 0.001
GB	Number of trees (100), Learning rate (0.1), Replicable training (Yes), Maximum depth (3), Fraction of training instances (1), Stop splitting subsets (2)
LR	Regularization (Ridge Regression (L2) with α = 0.0001), Fit intercept (Yes)
DT	Pruning (in leaves 2, internal nodes 5), Maximum depth (100), Splitting (95%), Binary trees (Yes)
SVM	C (1.0), ε (0.1), Kernel (RBF, exp(-auto|x-y|^2^)), Numerical tolerance (0.001), Iteration (100)
AdaB	Estimator (tree), Number of estimators (50), Algorithm (Samme.r), Loss (Linear Reg.)
kNN	Number of neighbors (5), Metric (Euclidean), Weight (Uniform)
SGD	Loss function (Huber), ε (0.1), Regularization (elastic net), Mixing (0.15), Learning rate (constant), Iteration (1000)

**Table 3:** The test performance results of the ML algorithms for the *x*-axis were evaluated based on the RCI, SFE, and a combination of RCI and SFE features.

Model	RCI			SFE			RCI + SFE		
	RMSE	MAE	R^2^	RMSE	MAE	R^2^	RMSE	MAE	R^2^
AdaB	0.290	0.044	0.999	1.018	0.156	0.988	0.344	0.055	0.999
GB	0.484	0.100	0.997	1.575	0.323	0.971	0.526	0.109	0.997
kNN	1.137	0.750	0.985	3.482	1.968	0.859	1.141	0.753	0.985
LR	1.600	1.200	0.970	8.320	7.108	0.196	1.577	1.192	0.971
ANN	0.627	0.383	0.995	4.926	3.631	0.718	0.528	0.313	0.997
RF	0.687	0.324	0.995	1.998	0.857	0.954	0.693	0.311	0.994
SGD	2.061	1.464	0.951	8.577	6.898	0.146	2.015	1.433	0.953
SVM	3.918	3.423	0.822	8.633	7.737	0.135	3.867	3.368	0.826
DT	0.777	0.190	0.993	2.361	0.643	0.935	0.769	0.196	0.993

**Table 4:** The performance of the ML algorithms for the *y*-axis was assessed using the RCI features, SFE features, and their combination.

Model	RCI			SFE			RCI + SFE		
	RMSE	MAE	R^2^	RMSE	MAE	R^2^	RMSE	MAE	R^2^
AdaB	0.470	0.063	0.999	1.311	0.207	0.989	0.369	0.058	0.999
GB	0.842	0.163	0.995	1.445	0.318	0.986	0.715	0.156	0.997
kNN	1.666	1.012	0.982	3.626	2.150	0.915	1.653	1.020	0.982
LR	2.433	1.837	0.962	8.648	6.888	0.517	2.412	1.817	0.962
ANN	1.213	0.750	0.990	5.939	4.575	0.772	1.027	0.590	0.993
RF	1.097	0.511	0.992	1.803	0.830	0.979	1.056	0.458	0.993
SGD	3.427	2.437	0.924	8.802	6.931	0.499	3.468	2.450	0.922
SVM	8.770	7.770	0.503	9.916	8.921	0.364	8.547	7.577	0.528
DT	1.153	0.299	0.991	2.261	0.644	0.967	1.125	0.288	0.992

**Table 5:** The test performance results of the ML algorithms for the *z*-axis were evaluated based on the RCI, SFE, and the combination of RCI and SFE features.

Model	RCI			SFE			RCI + SFE		
	RMSE	MAE	R^2^	RMSE	MAE	R^2^	RMSE	MAE	R^2^
AdaB	0.091	0.003	0.999	0.418	0.043	0.989	0.121	0.004	0.999
GB	0.356	0.059	0.992	0.442	0.084	0.988	0.320	0.058	0.994
kNN	0.711	0.228	0.969	1.905	0.941	0.777	0.700	0.229	0.970
LR	0.882	0.671	0.952	3.423	2.860	0.279	0.881	0.671	0.952
ANN	0.383	0.214	0.991	2.482	1.895	0.621	0.332	0.166	0.993
RF	0.432	0.152	0.989	0.652	0.237	0.974	0.412	0.139	0.990
SGD	1.272	0.959	0.900	3.476	2.810	0.257	1.257	0.925	0.903
SVM	2.457	2.119	0.629	3.493	3.114	0.249	2.341	2.061	0.663
DT	0.601	0.103	0.978	0.697	0.130	0.970	0.506	0.085	0.984

**Table 6:** The top seven features that exhibit the most significant impact on the AdaB algorithm’s performance, trained using dBm values and evaluated using the MAE and R^2^ metrics for the x, y, and z coordinates.

Feature Rank	x-Axis				y-Axis				z-Axis			
	MAE		R^2^		MAE		R^2^		MAE		R^2^	
1	${dBm}_{39}$	1.983	${dBm}_{39}$	0.173	${dBm}_{16}$	0.912	${dBm}_{0}$	0.058	${dBm}_{0}$	0.455	${dBm}_{0}$	0.112
2	${dBm}_{57}$	1.061	${dBm}_{57}$	0.078	${dBm}_{0}$	0.825	${dBm}_{16}$	0.038	${dBm}_{18}$	0.077	${dBm}_{18}$	0.016
3	${dBm}_{53}$	0.190	${dBm}_{63}$	0.004	${dBm}_{34}$	0.487	${dBm}_{34}$	0.019	${dBm}_{75}$	0.058	${dBm}_{75}$	0.012
4	${dBm}_{62}$	0.169	${dBm}_{53}$	0.003	${dBm}_{13}$	0.452	${dBm}_{13}$	0.014	${dBm}_{29}$	0.046	${dBm}_{29}$	0.009
5	${dBm}_{37}$	0.166	${dBm}_{62}$	0.003	${dBm}_{28}$	0.235	${dBm}_{28}$	0.006	${dBm}_{26}$	0.026	${dBm}_{79}$	0.003
6	${dBm}_{63}$	0.124	${dBm}_{94}$	0.004	${dBm}_{12}$	0.176	${dBm}_{33}$	0.003	${dBm}_{79}$	0.016	${dBm}_{26}$	0.003
7	${dBm}_{18}$	0.073	${dBm}_{37}$	0.003	${dBm}_{33}$	0.094	${dBm}_{12}$	0.002	${dBm}_{10}$	0.009	${dBm}_{10}$	0.001

**Table 7:** The test performance results of the AdaB algorithm for the x, y, and z axis were evaluated based on the dBm signal strength feature of the Wi-Fi signals.

	dBm (All)			RCI			dBm (7 Wi-Fi)			dBm (10 Wi-Fi)		
Axis	RMSE	MAE	R^2^	RMSE	MAE	R^2^	RMSE	MAE	R^2^	RMSE	MAE	R^2^
x	0.294	0.047	0.999	0.290	0.044	0.999	1.888	0.811	0.959	1.324	0.438	0.979
y	0.472	0.063	0.999	0.470	0.063	0.999	1.306	0.492	0.988	0.760	0.096	0.996
z	0.122	0.005	0.999	0.091	0.003	0.999	0.528	0.134	0.982	0.362	0.048	0.991

**Table 8:** Displays performance results when using the dBm values of well-performing Wi-Fi signals in the AdaB algorithm for the x, y, and z axes.

Axis	dBm (8 Wi-Fi) Wi-Fi: 0, 16, 18, 34, 39, 53, 57, 75			dBm (11 Wi-Fi) Wi-Fi: 0, 13, 16, 18, 29, 34, 39, 53, 57, 62, 75		
	RMSE	MAE	R^2^	RMSE	MAE	R^2^
**x**	1.05	0.52	0.987	0.58	0.17	0.996
**y**	2.55	1.27	0.957	0.68	0.1	0.997
**z**	1.29	0.5	0.898	0.4	0.041	0.99

**Table 9:** Comparison of Proposed Method with Existing Studies.

Refs.	No. Sample (Feature)	No. Locations	No. Access Points	Device Type(s)	Localization Area in m^2^	Performance in Meters	Assistive Technology
[47]	21,048 (529)	933	520	Smartphones	3 different building total: 108,703 m^2^	3D MAE: 6.05	No
[41,42]	~300 (NA)	32	NA	Raspberry Pi 3 and Arduino	Room 1: 6.0 × 5.5 m^2^ Room 2: 5.8 × 5.3 m^2^ Room 3: 10.8 × 7.3 m^2^	2D RMSE: 1.356 2D RMSE: 1.166 2D RMSE: 1.266	ZigBee and Bluetooth
[34,48,49,50]	365,000 (117)	345	6	Smartphone	21 × 16 m^2^	2D MAE: 0.75 m	No
[15]	23,925 (945)	1802	105	Smartphones	3 buildings 8000 m^2^	2D MAE: 2.3 m	No
Proposed Method	36,568 (312)	398	100	Raspberry Pi 4	1 building 3500 m^2^	2D RMSE: 0.552 3D MAE: 0.077	No

## Data Availability

The data presented in this study are available upon request from the corresponding author. (Please specify the reason for restriction, e.g., the data are not publicly available due to privacy or ethical restrictions.)
